# Enhanced artemisinin yield by expression of *rol* genes in *Artemisia annua*

**DOI:** 10.1186/s12936-015-0951-5

**Published:** 2015-10-29

**Authors:** Erum Dilshad, Rosa Maria Cusido, Javier Palazon, Karla Ramirez Estrada, Mercedes Bonfill, Bushra Mirza

**Affiliations:** Department of Biochemistry, Faculty of Biological Sciences, Quaid-i-Azam University, Islamabad, Pakistan; Laboratorio de Fisiologia Vegetal, Facultad de Farmacia, Universidad de Barcelona, Barcelona, Spain

**Keywords:** *Artemisia annua*, Artemisinin, *rol genes*, Hyb8001r, Trichomes

## Abstract

**Background:**

Despite of many advances in the treatment of malaria, it is still the fifth most prevalent disease worldwide and is one of the major causes of death in the developing countries which accounted for 584,000 deaths in 2013, as estimated by World Health Organization. Artemisinin from *Artemisia annua* is still one of the most effective treatments for malaria. Increasing the artemisinin content of *A. annua* plants by genetic engineering would improve the availability of this much-needed drug.

**Methods:**

In this regard, a high artemisinin-yielding hybrid of *A. annua* produced by the centre for novel agricultural products of the University of York, UK, was selected (artemisinin maximally 1.4 %). As *rol* genes are potential candidates of biochemical engineering, genetic transformation of *A. annua* with *Agrobacterium tumefaciens* GV3101 harbouring vectors with *rol B* and *rol C* genes was carried out with the objective of enhancement of artemisinin content. Transgenic lines produced were analysed by the LC–MS for quantitative analysis of artemisinin and analogues. These high artemisinin yielding transgenics were also analysed by real time quantitative PCR to find the molecular dynamics of artemisinin enhancement. Genes of artemisinin biosynthetic pathway were studied including amorphadiene synthase (ADS), cytochrome P450, (CYP71AV1) and aldehyde dehydrogenase 1 (ALDH1). Trichome-specific fatty acyl-CoA reductase 1(TAFR1) is an enzyme involved in both trichome development and sesquiterpenoid biosynthesis and both processes are important for artemisinin biosynthesis. Thus, real time qPCR analysis of the TAFR1 gene was carried out, and trichome density was determined.

**Results:**

Transgenics of *rolB* gene showed two- to ninefold (the decimal adds nothing in the abstract, please simplify to two- to ninefold) increase in artemisinin, 4–12-fold increase in artesunate and 1.2–3-fold increase in dihydroartemisinin. Whereas in the case of *rol C* gene transformants, a fourfold increase in artemisinin, four to ninefold increase in artesunate and one- to twofold increase in dihydroartemisinin concentration was observed. Transformants with the *rol B* gene had higher expression of these genes than *rol C* transformants. TAFR1 was also found to be more expressed in *rol* gene transgenics than wild type *A. annua*, which was also in accordance with the trichome density of the respective plant.

**Conclusion:**

Thus it was proved that *rol B* and *rol C* genes are effective in the enhancement of artemisinin content of *A. annua*, *rol B* gene being more active to play part in this enhancement than *rol C* gene.

## Background

Artemisinin, a highly potent anti-malarial agent found in the medicinal plant *Artemisia annua*, is part of the artemisinin-based combination therapy recommended by the World Health Organization for the treatment of malaria [1, 2]. Besides malaria, artemisinin is also effective against some viral and parasitic diseases i.e. hepatitis B, schistosomiasis, chagas disease and African sleeping sickness. It is also effective in the reduction of angiogenesis and treatment of some cancers [3]. Owing to the fact that availability of artemisinin is insufficient to meet worldwide demand, efforts are needed to increase its quantity. In this regard, some improvement has taken place by the Artemisia research project of CNAP (Centre for Novel Agricultural Products, University of York, UK). Their breeding programme has developed some high artemisinin-producing hybrids by making crosses between parental lines with consistent field performance, high artemisinin content and high seed productivity. Two hybrids which have been released commercially include Hyb1209r (Shennong, artemisinin 1.5 %) and Hyb8001r (Zenith, artemisinin 1.4 %) [4].

Artemisinin synthesis occurs in the glandular trichomes (GLTs) present on flowers, floral buds and leaves. Trichome-specific fatty acyl-CoA reductase 1 (TAFR1) is thought to be involved in GLT development and sesquiterpenoid biosynthesis; which are important for artemisinin production [5, 6]. Other enzymes like amorphadiene synthase (ADS), cytochrome P450, CYP71AV1 (CYP), double bond reductase 2 (DBR2) and aldehyde dehydrogenase 1 (ALDH1) catalyze different steps of artemisinin biosynthesis [7–11]. Overexpression of these enzymes may lead to increased artemisinin accumulation and also the derivatives. Some studies provide evidence that metabolic engineering of the biosynthetic pathway that leads to artemisinin by the insertion of different genes can increase artemisinin content *in planta* [12, 13]. In this regard, *rol* genes of *A. rhizogenes* are considered to be effective inducers of secondary metabolites production in plants [14]. *Rol A* is a DNA binding protein and stimulator of growth, *rol B* having tyrosine phosphatase activity regulates signal transduction pathway of auxin [15, 16], and *rol C* has cytokinin glucosidase activity. However, each gene seems to affect plant morphology and stimulate production of different secondary metabolites [17–20]. However, there is no report about production of transgenic *A. annua* plants with individual *rol* genes for enhancement of artemisinin and its derivatives. Previous work on transformation of other *Artemisia* sp. with *rol* genes showed not only over expression of artemisinin biosynthetic genes, but also artemisinin in the plant [21].

In the continuation of that work, genetic transformation of *A. annua* (Hyb8001r, Zenith) with *rol* genes (*rol B* and *rol C*) was carried out and consequences on the content of artemisinin were evaluated by LC–MS. The real time qPCR analysis of the genes involved in biosynthetic pathway of artemisinin (ADS, CYP71AV1 and ALDH1) provided an understanding of the molecular mechanism through which *rol* genes can enhance the artemisinin production. Expression of the gene for TAFR1 was also found to be relevant in this respect, providing evidence for molecular dynamics of artemisinin accumulation pattern and correlating it with the expression of genes involved in its biosynthesis.

## Methods

### Seeds germination and DNA barcoding

Seed of *A. annua* were purchased from East West seeds international company (Thailand). After collection and surface sterilization with 70 % ethanol, they were germinated on half strength MS medium. Genomic DNA of germinated plantlets was extracted according to reported protocol [22]. Non-coding spacer region between the *psbA* and *trnH* genes of chloroplast DNA was amplified by PCR for the identification of *A. annua*, using primers of *psbAtrnH* region given in Table 1. The PCR reaction was carried out according to the reaction conditions reported [23]. PCR products were purified by Rapid PCR Purification System 9700 (Marligen Biosciences, Ijamsville, MD, USA), which was then sequenced following dideoxy-chain termination method using an ABI Prism 310 Automated DNA Sequencer (PE, Applied Biosystems, Foster City, CA, USA). Sequences were identified and aligned via BioEdit sequence alignment tool (editor version 7.2.5.0).

**Table 1:** Accession numbers and primer sequences of the genes studied

S. no	Gene name	Primer sequences	Accession no
1	psbA-trnH	Forward: GTTATGCATGAACGTAATGCTC-3 Reverse: CGCGCATGGTGGATTCACAATC	FJ418749
2	Rol B	Forward: GCTCTTGCAGTGCTAGATTT Reverse: GAAGGTGCAAGCTACCTCTC	X15952.1
3	Rol C	Forward: GAAGACGACCTGTGTTCTC Reverse: CGTTCAAACGTTAGCCGATT	X64255.1
4	ADS	Forward: ATTACTGGCGGTGCTAAC Reverse: GTGCAGAGACAGCCCATT	JQ319661
5	CYP71AV1	Forward: ATTTTGGATATGTTTGGAGCAGGC Reverse: TCCGCTTGTACTTTCTCCATTGCT	HQ315834
6	ALDH1	Forward: CAGGAGCTAATGGAAGTTCTAAGTCAG Reverse: TTTCTTCCTTCGGCCACTGTTG	FJ809784
7	TFAR1	Forward: CCTTGGAGATCCTGAAGCTG Reverse: CGTTGGATTGTGCTGAACTG	GU733320
8	Actin	Forward: ATCAGCAATACCAGGGAACATAGT Reverse: AGGTGCCCTGAGGTCTTGTTCC	EU531837
9	GADPH	Forward: TGGGATCGTTGAGGGTCTT Reverse: GCTGCTGGGAATAATGTTGAA	GQ870632.1

### Bacterial strains and plasmids

**Fig. 1:**
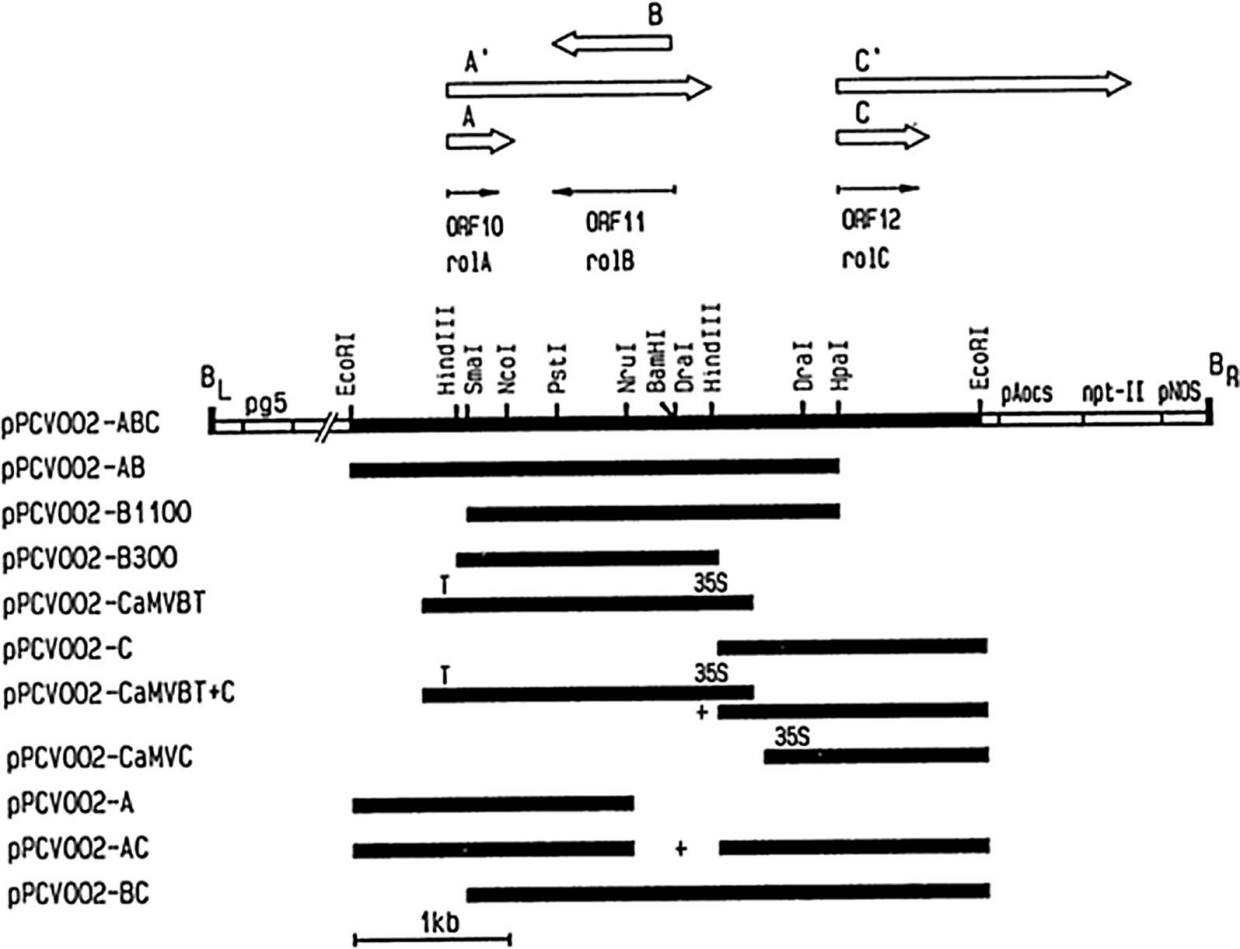
Detail of vectors used for transformation of *Artemisia annua*: Vectors (pPCV002-CaMVBT and pPCV002-CaMVC) used in the transformation of *Artemisia annua* (Fig. taken from Spena et al. [24])

*Agrobacterium tumefaciens* strain GV3101 containing plasmid pPCV002-CaMVBT and pPCV002-CaMVC kindly provided by Dr. A. Spena, Max-Planck-Institut fur Zuchtungsforschung, 5000 Koin 30, FRG [24] were used for transformation purpose. The T-DNA region of the plasmid pPCV002-CaMVBT contained the coding sequence of *rol* B gene and that of pPCV002-CaMVC contained coding region of *rol* C gene, which were under the control of CaMV35S promoter. T-DNA of pPCV002-CaMVBT and pPCV002-CaMVC also contained the *neomycin phosphotransferase* (*NPTII*) gene with nopaline synthase (NOS) promoter and NOS terminator sequences (Fig. 1). *A. tumefaciens* containing the plasmids pPCV002-CaMVBT and pPCV002-CaMVC were grown overnight in Luria broth (Sigma Cat # L-1900). After inoculation, bacterial cultures were maintained at 28 °C and 120 rpm in a shaking incubator. Growth was obtained in 24 h and OD was checked by spectrophotometer at 600 nm, when in the range of 0.2–0.8, the bacterial suspension was used for the transformation purpose.

### Transformation and confirmation of transgene integration

For transformation purpose 1 month-old in vitro grown plants were used. Nodal explants were prepared and after preculturing of 2 days on shooting media (2.5 mg/l BAP and 0.25 mg/l NAA) supplemented with 200 µM acetosyringone, they were given the infection with bacterial strains containing the desired constructs. Explants were given bacterial infection for 10–15 min afterwards explants were dried on autoclaved filter paper for 2–3 min and then placed on MS shooting media supplemented with 200 µM acetosyringone. After 2 days of incubation in dark at 28 °C, explants were washed with antibiotics and placed on selection medium (2.5 mg/l BAP, 0.25 mg/l NAA, 50 mg/l kanamycin, 300 mg/l cefotaxime). Regeneration occurred within 1 month, explants were shifted to fresh selection media after every 20 days. After getting the shoots of considerable, length they were shifted to the rooting media (0.1 mg/l NAA, 50 mg/l kanamycin) Three to four cycles of selection were completed until complete plant was regenerated on selection media.

Molecular analysis was performed after extraction of genomic DNA from shoots of 2 month-old transformed and wild type plants by the CTAB method [22]. The plasmid from GV3101 was isolated by alkaline lysis method. PCR analysis was performed using a programmed DNA thermal cycler (Biometra, USA). The primers of *rol B* and *rol C* gene are given in Table 1. Conditions applied for PCR were according to a previous report [21].

### Analysis of artemisinin and its derivatives by HPLC-ESI/MS

Extraction of artemisinin and its derivatives from leaves of 4–5 month old transformed and wild type plants was carried out with a previously published method [25]. HPLC–MS analysis was performed with Varian 500 Ion Trap Mass spectrometer (Varian, Spain) coupled to a HPLC system equipped with auto sampler (Varian, Spain). Separation of the analytes was achieved by C 18 (5 µm) column (150 mm × 4.6 mm) (AKDAY Chromatografica, Spain) using mobile phase consisting of water with 0.1 % formic acid (A) and acetonitrile (B) in a gradient as follows: [t (min), % B] (0, 50) (10, 50) (15, 99) (23, 50) (26, 50). The flow rate was 1 ml/min. Electrospray ionization (ESI) in positive mode was applied with a capillary voltage of 4000 V and plate voltage of 600 V. Other parameters include: spray chamber temperature 50 °C, nebulizing gas (N_2_) pressure: 35 psi, dry gas (N_2_) 10 L/min at 35 psi and 400 °C. The mass analyzer scanned from 66 to 500 u. Fragmentation amplitude was 1.0 V.

### Expression analysis of *rol* genes and the genes related to artemisinin biosynthesis

Total RNA from leaves of transformed and wild type plants (4–5 months old) was extracted according to a previously published procedure [26]. Turbo DNAse (Ambion) was used to ensure complete removal of DNA from RNA after extraction. The purity and quantity was checked by taking absorbance at 260 and 280 nm on Nanodrop ND-2000 spectrophotometer (Thermoscientific). The quality of RNA was also assessed by running RNA samples on 1.2 % agarose gel. Reverse transcription of 1 μg of RNA was carried by using 1st strand cDNA synthesis kit (Invitrogen) following the manufacturer’s instructions. In order to check the expression of *rol* B and *rol* C gene, semi-quantitative reverse transcriptase-polymerase chain reaction was performed with *rol* B and *rol* C gene primers as done previously by using 1 μl of cDNA reaction mixture as template. The gel image of PCR products were scanned by Kodak Molecular Imaging software (version 4.2) and integrated density values were calculated which were found to be different for each band.

To evaluate the possible effects of *rol* genes on the expression of artemisinin biosynthetic genes, quantitative real time PCR of four selected genes was performed, namely those encoding amorpha-4, 11 diene synthase (ADS), cytochrome P450 (CYP71AV1) and aldehyde dehydrogenase 1 (ALDH1). The gene involved in trichome development and sesquiterpenoid biosynthesis (TFAR1) was also studied. The β-actin gene was used as reference gene [27]. For real time qPCR, a 1:4 dilution of cDNA was used. The amplification reaction was performed by gene specific primers as given in Table 1; primer amplification efficiency was determined as previously described [28]. The qPCR was performed using iTAqTM Universal SYBR Green Supermix (BioRad, Hercules, CA, USA) in a 384-well platform system (ABI Prism^®^ 7900HT sequence detection system, Applied Biosystems, Foster, CA, USA). The reaction conditions for real time qPCR were as follows: denaturation for 5 min at 95 °C, followed by 45 cycles each of denaturation for 10 s at 95 °C, annealing for 10 s at 60 °C, followed by elongation for 10 s at 72 °C. For each gene, the relative expression levels were normalized with respect to the wild-type plant (reference value = 1).

### Calculation of trichome density

The leaves of wild type and transformed plants of *A. annua* were selected for the calculation of trichome density. For that purpose the ventral surfaces of the leaves were adhered to microscope slides with the help of glue stick and placed overnight at 4 °C for dehydration. The next day, slides were examined under the fluorescent microscope (Leica) with a FITC green filter. Slides were studied under 10× resolution and photographed.

### Statistical analysis

All the experiments including, PCR, LC–MS and trichomes analysis were run in triplicate. Results of metabolites content, real-time quantitative PCR analysis and calculation of trichome density are presented as mean values ± SE. All the data obtained for the qualitative and quantitative analysis of artemisinin and derivatives, were statistically analysed by ANOVA and Duncan’s multiple range test using Mstat C software. Statistical significance of trichomes was determined by t test (**, P < 0.01; *, P < 0.05).

## Results and discussion

### Plant identification through DNA barcoding

Amplification of 500 bp *psbA*-*trnH* region of the chloroplast genome was successfully carried out. DNA samples were sequenced in triplicate to confirm the authenticity of species-specific nucleotides and got the same results. Gene bank accession number [NCBI: FJ418749] (*A. annuapsbA*-*trnH* sequence) are reference sequence to confirm the plant species. After performing the CLUSTAL W in BioEdit software and the BLAST in NCBI, *psbA*-*trnH* sequence of *A. annua* was confirmed. There was 99.8 % similarity between standard and analysed sequence with a single base pair difference at 330 bp position where cytosine was observed instead of adenine. Similar results were obtained by another group that amplified the *psbA*-*trnH* region of *A. annua* under same conditions [23].

### Transformation and confirmation of transgene integration

**Fig. 2:**
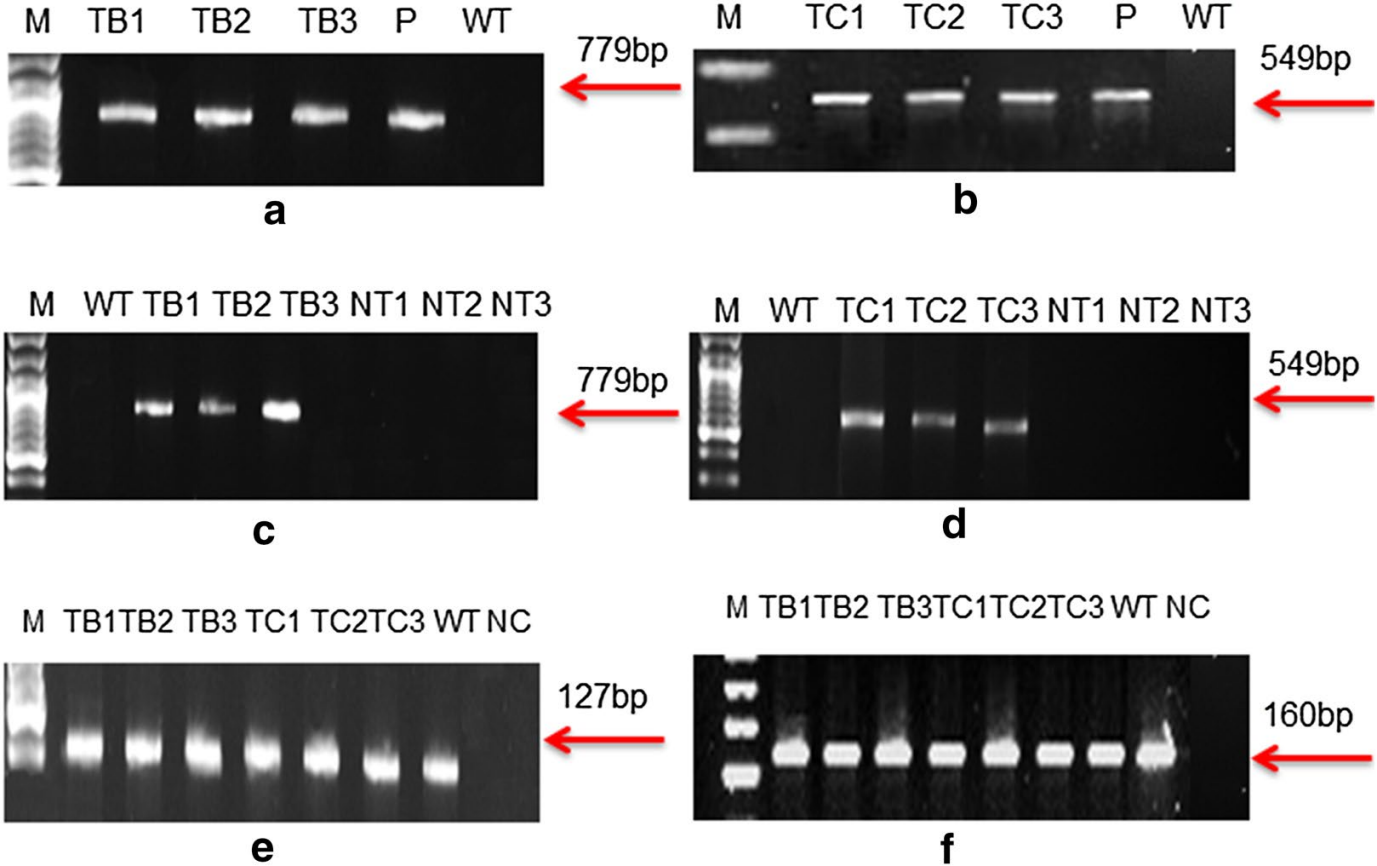
Gel pictures showing results of normal and semiquantitative reverse transcriptase PCR: PCR amplified products of *rol* B (**a**), *rol* C (**b**) gene are shown in the figure. Semiquantitative RT-PCR showing the relative expression of *rol* B (**c**) and *rol* C gene (**d**). *GADPH and β*-*actin* used as housekeeping gene (**e**, **f**). TB1–TB3 represents *rol* b transgenics whereas TC1–TC3 represent *rol* C transgenics. *WT* stands for wild type plant of *A. annua*, *lane P* refers to the plasmid DNA and *lane M* corresponds to the marker DNA (Fermentas). *NT* indicates the negative or “no RT control” for each transgenic line

Successful transformation of *A. annua* with *A. tumefaciens* GV3101 harbouring the *rol* B and *rol* C gene was carried out. Single transformation experiment was carried out, and 300 explants were used for each gene. Transformation efficiency was 30 %, calculated on the basis of plants showing PCR positive results with respect to total no of infected explants. Due to contaminating factors like fungal and bacterial, only three transformants of *rol* B and three transformants of *rol* C survived until maturity on selection media. PCR performed for the *rol* gene transformants of *A. annua* showed the amplified products of 779 bp for *rol* B, 549 bp for *rol* C gene, as shown in Fig. 2a, b. Similar amplified products were obtained from plasmid DNA of GV3101-CaMVBT and GV3101-CaMVC respectively. The genome of wild type plants did not contain these genes.

**Fig. 3:**
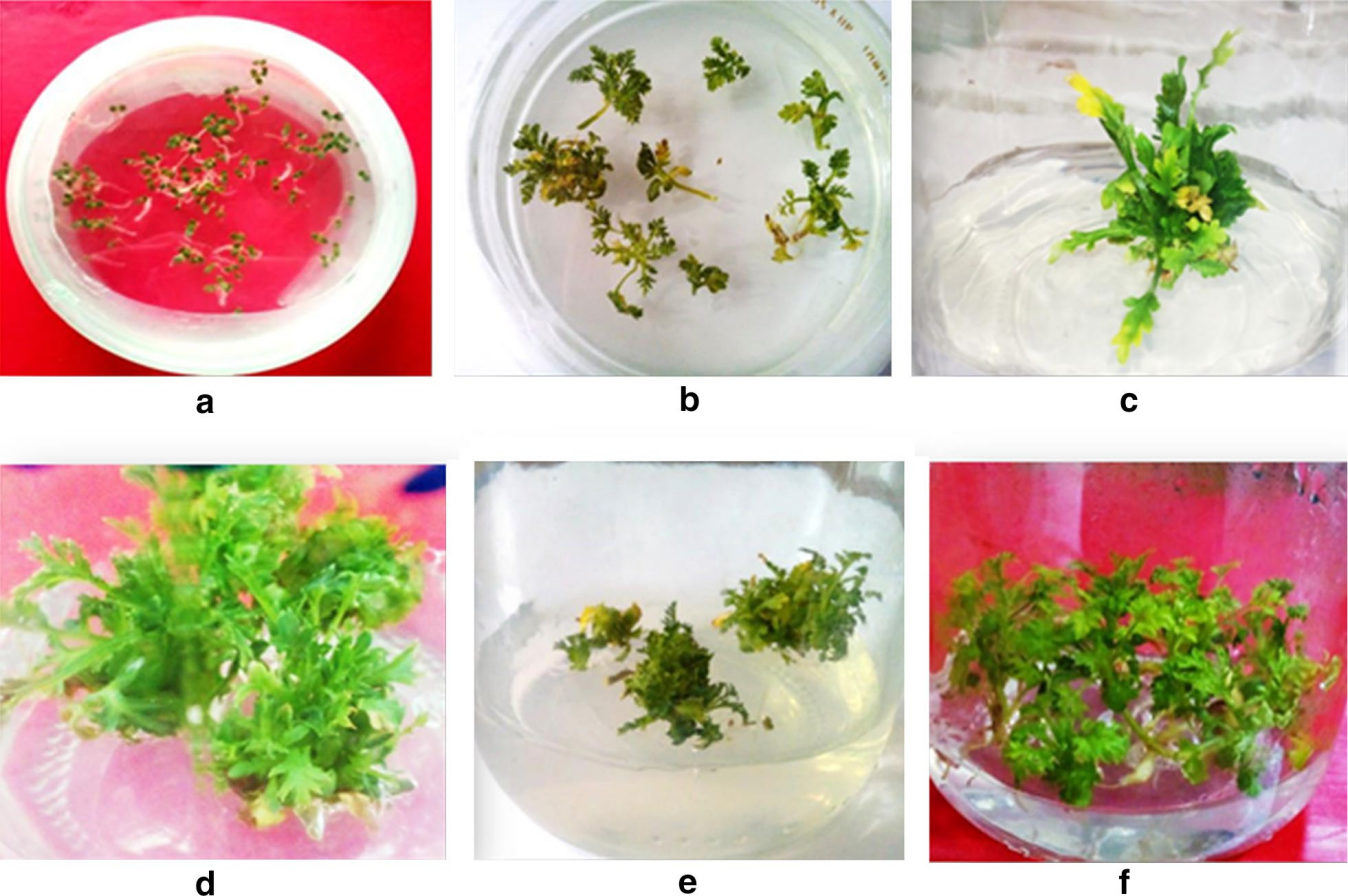
Vegetative propagation of *Artemisia annua*: seeds germination (**a**), explants after co-cultivation with bacteria (**b**), regeneration on selection media (**c**), tranformant of *rol* B (**d**), transformant of *rol* C gene (**e**) and wild type *A. annua* (**f**)

Morphological variability was distinct among transgenics and wild type *A. annua*. *Rol* B transgenics grew faster on the selection media with altered morphology showing increased inflorescence, while transgenics of *rol* C gene had totally different morphology with an altered leaf phenotype showing narrow leaf blade and decreased internode length (Fig. 3). It is reported that morphological variations can be due to the presence of *rol* genes transcripts and can be attributed to the position effect [29]. Several papers report variation in morphological characteristics of various plants species due to *rol* gene integration [30–32]. In fact, enhanced sensitivity of *rol B* transformed cells toward auxin caused by increased tyrosine phosphatase activity of *rol B* gene can result in altered phenotype of *rol B* transgenics [33, 34]. Apart from this, H^+^ATPase and ionic imbalance [33] also synchronize the metabolic pathways leading to phenotypic variations [35]. *Rol C* is involved in functional imbalance of polyamines, gibberellins, auxins, and cytokinins [36, 37]. Such imbalances in plant growth hormones can alter plant root and shoot phenotypes [35]. However, there was no effect on the overall growth rate of shoots as confirmed by measuring the fresh and dry weight of the wild type and transformed plants.

### Analysis of artemisinin and derivatives by LC–MS

**Fig. 4:**
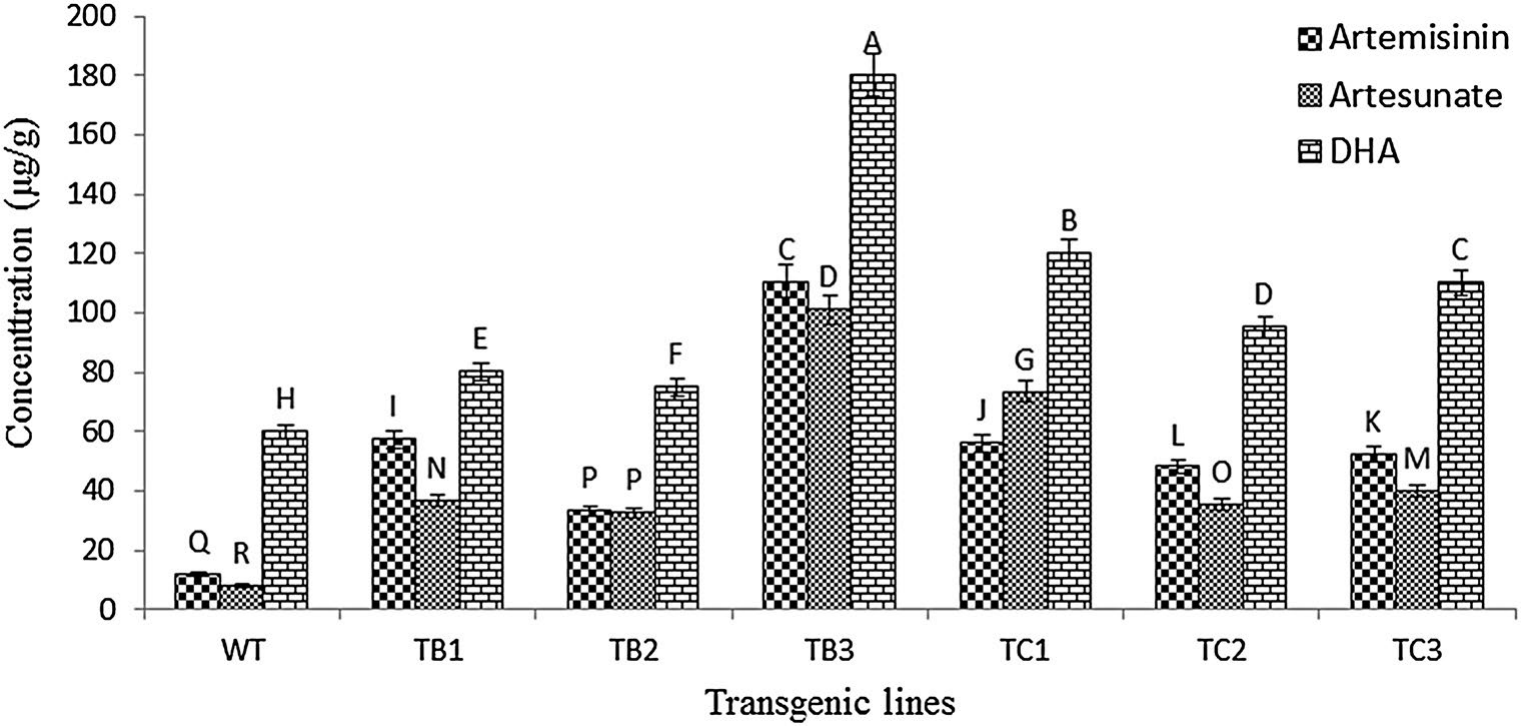
Statistical and quantitative analysis of artemisinin and derivatives: comparative and statistical analysis of artemisinin content and its derivatives in wild type *A. annua* and transgenics of *rol* B and *rol* C gen. *Letters* on the *bars* show LSD ranking

Differences were observed in the content of artemisinin and derivatives in transformed and untransformed plants. All of the transgenic lines of *rolB* and *rol C* gene showed enhancement of artemisinin and derivatives (Fig. 4). Transgenics of *rolB* gene showed 2.7–9.2-fold increase in artemisinin, 4–12.6-fold increase in artesunate and 1.2–3-fold increase in D.H.A. Whereas in case of *rol C* gene transformants 4–4.6-fold increase in artemisinin, 4.4–9.1-fold increase in artesunate and 1.5–2-fold increase in D.H.A concentration was observed. Previously it is reported that *rol* genes (*rol A, rol B* and *rol C*) when expressed individually or combined result in increased plant secondary metabolism by transcriptional activation of defense genes through an unknown mechanism [38]. The effects of individual *rol* genes of TL-DNA of *Agrobacterium rhizogenes*, A4 strain on production of ginsenoside of *Panax ginseng* cell cultures has been reported [17]. In this report, *rolC* cultures accumulated 1.8–3 times more ginsenoside than control plant [17]. *Ro*l *B* transgenics of *Rubia cordifolia* showed enhanced production of anthraquinones [38]. Further, *rolB* gene from *A. rhizogenes* when expressed in tomato improved foliar tolerance against fungal pathogens [39]. However, in the current study differences in artemisinin content of *rol B* and *rol C* transgenics were observed. *Rol B* gene was found to be more active in the enhancement of artemisinin content than *rol C* gene. As previously reported, high expression of the *rol B* gene dramatically increased the biosynthesis of secondary metabolites in transformed plant cells [38]. Compared to the *rol B* gene, the *rol C* gene activated the biosynthesis of secondary metabolites to a lesser extent. Evidence indicates that each of the *rol* genes has its own role in plant metabolic processes [14]. Transgenic plants showing increased artemisinin concentration and altered morphology were analysed by semi quantitative RT-PCR to check that whether the results correlate with *rol* gene expression. Results confirmed the findings to be the result of presence of *rol* gene transcripts. Also it was observed that the lines TB3 and TC1 had higher artemisinin content and more transcripts of *rol* B and *rol* C gene, respectively (Fig. 2c, d). As these genes are under the control of CaMV35S promoter, their effect on the induction of secondary metabolism become more clear due to systemic high level of expression [40].

### Expression analysis of artemisinin and trichome biosynthesis genes

**Fig. 5:**
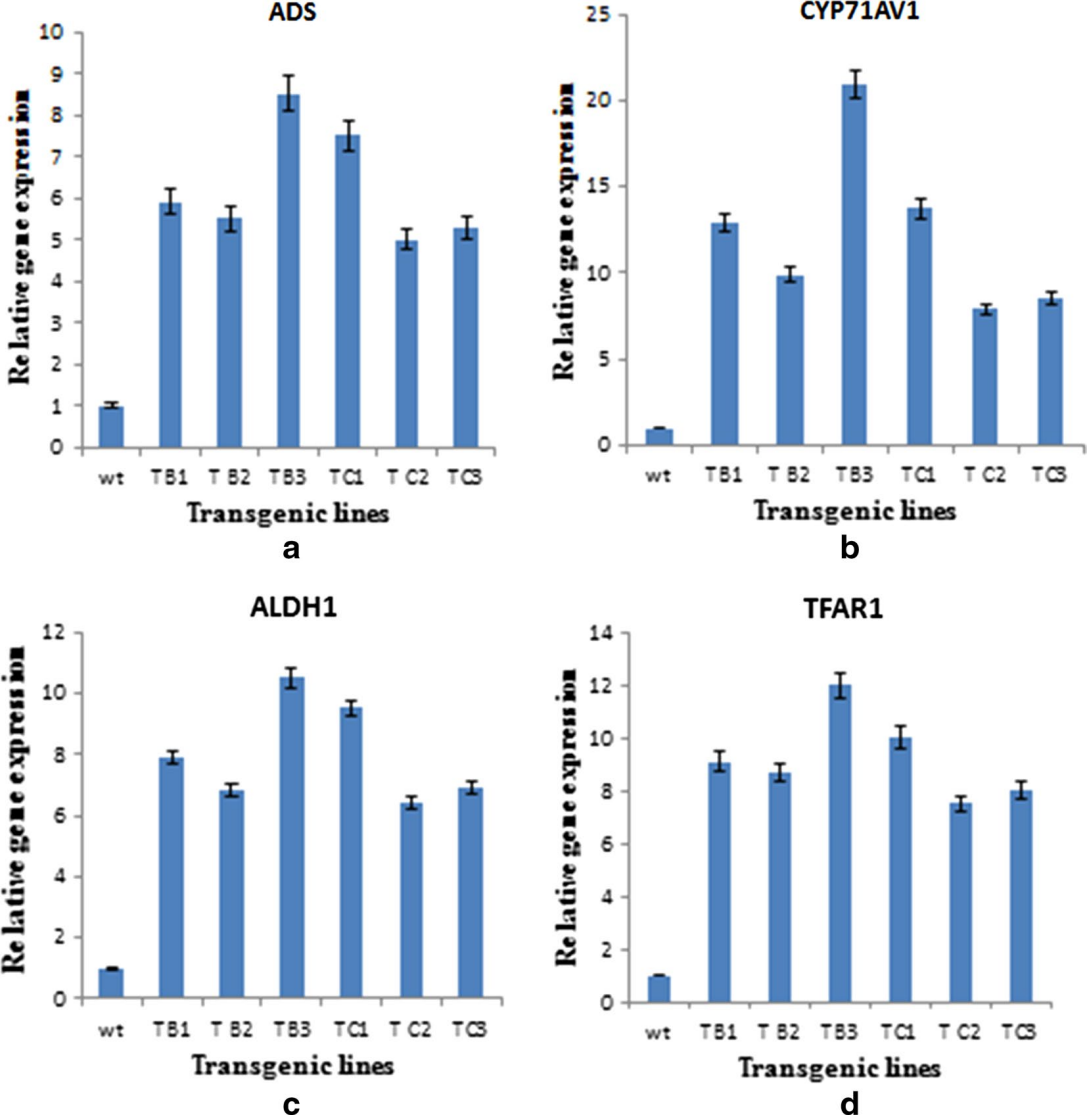
Expression analysis of artemisinin biosynthetic pathway genes and TAFR1 gene: expression level of artemisinin biosynthetic genes, ADS (**a**), CYP71AV1 (**b**) and ALDH1 (**c**) in wild type plant of *A. annua* and transgenics of *rol B* and *rol C* gene. TFAR1 is the gene involved in trichome development and sesquiterpenoid biosynthesis (**d**)

Relatively higher transcripts of all studied genes of artemisinin biosynthetic pathway were found in the transgenics of *rol* B and *rol* C gene although in variable fashion (Fig. 5), which also correlates the accumulation pattern of artemisinin and analogues. Regarding the biosynthesis of artemisinin, ADS, CYP71AV1 and ALDH1 are key genes involved in the conversion of FDP (Farnesyl diphosphate) to artemisinic acid which is a late precursor of artemisinin, as discussed in the background section [7–11]. Transgenics of *rolB* and *rolC* gene showed overexpression of studied genes with CYP71AV1 found to be most expressed in transgenics with 8–20.9-fold increase in *rol* B tranformants and 6–15.5-fold more expression in *rol* C transformants. ALDH1 gene showed eight- to tenfold and ADS gene showed 5.9–8.5-fold more expression in *rol* B transformants. Similarly Transgenics of *rol C* gene showed 6.4–9.5-fold increase in ALDH1 and 5–7.5-fold increase in ADS gene expression. Transgenic line TB3 and TC1 were found with highest expression of these genes. This relative expression of all the genes is in accordance with the accumulation of artemisinin and derivatives in respective transgenic lines. These results are supported by a previous report which demonstrates that overexpression of farnesyl pyrophosphate synthase (FPS) in *A. annua* increases the accumulation of artemisinin through conversion of isopentenyl diphosphate (IPP) and dimethylallyl diphosphate (DMADP) into farnesyl diphosphate (FDP) [41]. In another report, increased amount of artemisinin in *A. annua* transformed with *A. tumefaciens* wild type nopaline strains was observed [42]. Similarly, another group transferred the *Ipt* gene into *A. annua* and observed increased amount of artemisinin in transformed plants [13].

### Trichome density and expression of TFAR1 gene

**Fig. 6:**
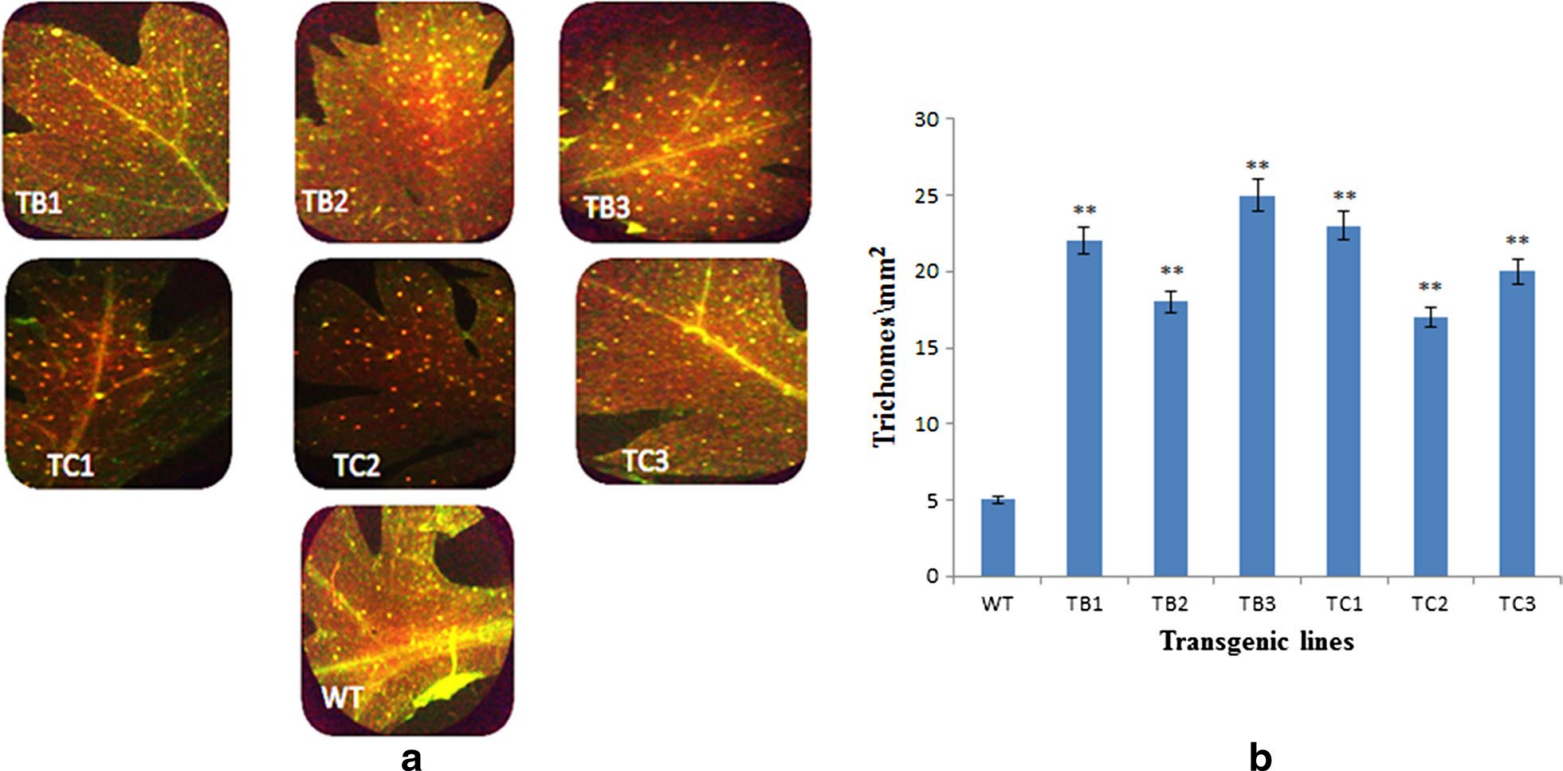
**a** Trichome density: comparison of trichome density of transformed and untransformed plants of *A. annua*. TB1–TB3 represent *rol B* transgenics whereas TC1–TC3 represent transgenics of *rol C* gene. *WT* represents the control plant of *A. annua.* **b** Graphical representation of trichomes density of wild type *A. annua* and transformants of *rol B* and *rol C* gene

The number of glandular trichomes was determined at the adaxial side of three random pieces of fresh leaf material from each sample with an accurately determined leaf area of approximately 1 cm^2^ per leaf and significant differences were observed as shown by Fig. 6. The leaves of *A. annua* transformed with *rol B* and *rol C* gene produced more trichomes (20–30 trichomes/cm^2^) as compared to untransformed leaves (5–7 trichomes/cm^2^).

TFAR1, which stimulates trichome development and catalyzes sesquiterpenoid biosynthesis [41] also showed increased expression in all transgenic lines. *Rol* B transformants with 2.3–3-fold increase and *rol* C gene transformants with 1.6–2-fold increase in TFAR1 gene expression were found. As mentioned in a previous report that glandular trichomes are the sites of production of important phytochemicals including artemisinin and a mixture of chemicals that have been found to have an enormous array of uses in the pesticides, pharmaceutical and flavor industries [43, 44]. Artemisinin content was found to be directly related to the trichome index in previous reports [45, 46].

## Conclusion

Transformation of *A. annua* with *rol* B and *rol* C gene results in the enhancement of its secondary metabolites particularly the artemisinin and derivatives, *rol B* gene being more active to play part in the enhancement of artemisinin than *rol C* gene. Further the level of transcripts of the *rolB* and *rolC* gene found in transgenics also correlate with their artemisinin accumulation pattern. Altered expression of genes involved in biosynthesis of artemisinin and trichomes was observed.

## Abbreviations

ADS: amorpha diene synthase
ALDH1: aldehyde dehydrogenase 1
CTAB: cetyl trimethylammonium bromide
CNAP: Centre for Novel Agricultural Products
CYP71AV1: cytochrome P450
DBR2: double bond reductase 2
D.H.A: dihydroartemisinin
DMADP: dimethyl diphosphate
FDP: farnesyl diphosphate
FPP: farnesyl pyrophosphate
GADPH: glyceraldehyde-3-phosphate dehydrogenase
GLTs: glandular trichomes
IPP: isomethyl diphosphate
TAFR1: trichome specific fattyacyl CoA reductase 1
